# Reconstructing molar growth from enamel histology in extant and extinct *Equus*

**DOI:** 10.1038/s41598-017-16227-2

**Published:** 2017-11-21

**Authors:** Carmen Nacarino-Meneses, Xavier Jordana, Guillem Orlandi-Oliveras, Meike Köhler

**Affiliations:** 1 Institut Català de Paleontologia Miquel Crusafont (ICP), Campus de la Universitat Autònoma de Barcelona, 08193 Bellaterra, Barcelona, Spain; 2 Unitat d’Antropologia Biològica, BABVE department, Universitat Autònoma de Barcelona (UAB), 08193 Bellaterra, Barcelona, Spain; 3 ICREA, Pg. Lluís Companys 23, 08010 Barcelona, Spain

## Abstract

The way teeth grow is recorded in dental enamel as incremental marks. Detailed analysis of tooth growth is known to provide valuable insights into the growth and the pace of life of vertebrates. Here, we study the growth pattern of the first lower molar in several extant and extinct species of *Equus* and explore its relationship with life history events. Our histological analysis shows that enamel extends beyond the molar’s cervix in these mammals. We identified three different crown developmental stages (CDS) in the first lower molars of equids characterised by different growth rates and likely to be related to structural and ontogenetic modifications of the tooth. Enamel extension rate, which ranges from ≈400 μm/d at the beginning of crown development to rates of ≈30 μm/d near the root, and daily secretion rate (≈17 μm/d) have been shown to be very conservative within the genus. From our results, we also inferred data of molar wear rate for these equids that suggest higher wear rates at early ontogenetic stages (13 mm/y) than commonly assumed. The results obtained here provide a basis for future studies of equid dentition in different scientific areas, involving isotope, demographic and dietary studies.

## Introduction

The reconstruction of tooth growth is essential to understanding the biology and palaeobiology of mammals^1,2^, as dental development is closely related with a species’ life history^2–6^. For instance, the eruption of the first permanent molar correlates well with weaning^4,7^. Similarly, the emergence of the third molar correlates with skeletal maturity^2^. Thus, the estimation of rate and duration of molar growth in extant and extinct vertebrates yields key information about their pace of life^1–3,8–11^. Furthermore, an understanding of tooth growth is crucial for palaeoecological and palaeobiological studies that involve the analysis of stable isotopes^12,13^. Tooth crowns preserve a temporal record of isotopic variation that can be related to changes in climatic conditions and/or modifications of an animal’s behaviour^14–18^. Therefore, the rate and duration of tooth growth must be precisely known to accurately develop isotopic sampling methods^19^ and correctly interpret the isotopic data obtained in this type of investigation^12,13^.

The pace of growth and development of teeth is recorded, among others, in dental enamel^20^. From the cusp to the root, enamel is rhythmically deposited by enamel-forming cells called ameloblasts in the amelogenesis process, which involves a first stage of enamel secretion followed by a second phase of enamel maturation^21,22^. As a result, the histological microstructure of dental tissue registers the pattern of enamel growth in the form of incremental markings^20,23^. Incremental features have traditionally been classified as short- or long-period marks^24^. The first ones include cross-striations and laminations and represent a circadian rate of enamel formation^23,25,26^. Retzius lines, on the other hand, are long-period lines that indicate the successive positions of the developing enamel front^20,23^. Counts and measurements of incremental markings in enamel provide the basis for quantifying tooth growth, estimating various dental growth parameters such as daily secretion rate or extension rate, and for calculating crown formation time^1^.

In large herbivorous mammals, studies aimed at reconstructing tooth growth through the analysis of enamel incremental markings have increased considerably in number over the last years^3,8–11,19,27–29^. However, an analysis of life history parameters from the enamel microstructure in key groups of evolution such as equids^30^ is still lacking. Only the work of Hoppe *et al*.^13^ provided some data about the periodicity and disposition of incremental lines in the enamel of the domestic horse, but recently their results have been questioned by other authors^28,31^. Indeed, the development of equid teeth has hitherto been determined from radiographic observations^13,32–34^ or by measurements of the crown height^12,35^, but it has not yet been studied using dental histology.

In the present study, we aim to analyse the enamel microstructure of several wild equid species to provide information about the dental growth pattern and development in this mammalian group. Nowadays, the genus *Equus* comprises the wild extant species of zebras (*E*. *zebra*, *E*. *grevyi*, *E*. *quagga*), asses (*E*. *africanus*, *E*. *kiang*, *E*. *hemionus*) and horses (*E*. *ferus*) that dwell in different areas of Africa and Asia^36,37^. Here, we quantified incremental markings and enamel growth parameters in the Asiatic wild ass (*E*. *hemionus*), plains zebra (*E*. *quagga*) and Grevy’s zebra (*E*. *grevyi*). These three taxa are the most appropriate ones to infer the dental growth pattern of the clade, as they cover most of the range of habitat, body mass and life history traits observed in extant wild equids^38–42^. To apply our results to the equid fossil record, we examined the dental enamel of Pleistocene fossil specimens of *E*. *ferus* and *E*. *hydruntinus* to infer their pattern of molar growth. These two different-sized equids are frequent in European Late Pleistocene mammal assemblages^43^. Because body size is a fundamental life history trait that tightly correlates with other biological traits such as growth rate^44–46^, we calculated their molar growth rate as a proxy of their overall growth rate to understand whether the differences in body size resulted from changes in life history.

## Results

### Dental histology of extant *Equus*

**Figure 1:**
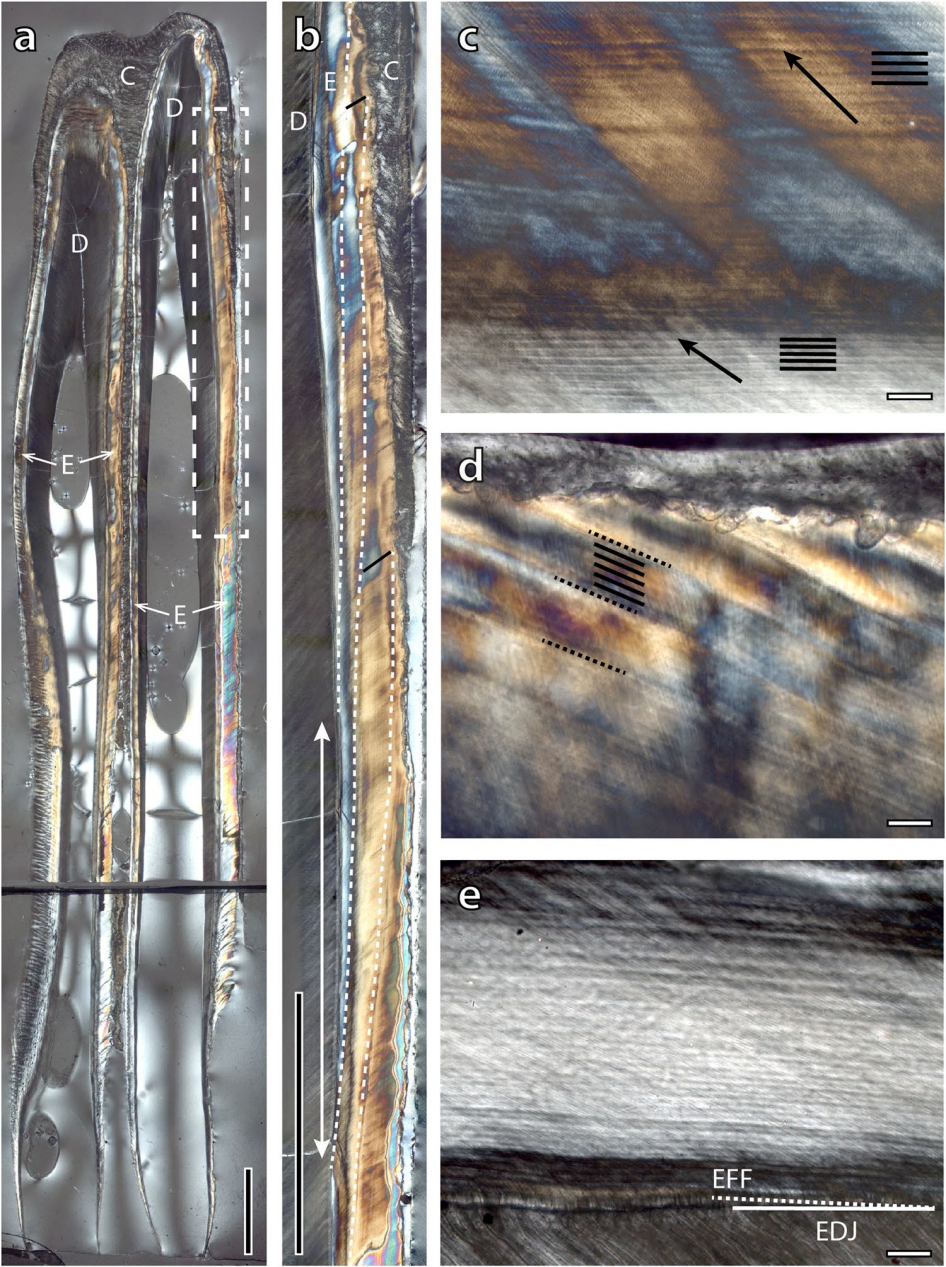
Dental histology of *Equus* and methodologies used to analyse the pattern of enamel growth. (**a**) Longitudinal section of *Equus hemionus*’ first lower molar (IPS83151) mounted on two different slides. White dashed rectangle indicates the magnified area in b. (**b**) Methodology employed to calculate the crown formation time (CFT; IPS83151). The distance between incremental lines (white dashed lines) is measured following the course of enamel prisms (black lines) and divided by the daily secretion rate to determine the time required to form a specific portion of the enamel dentine junction (EDJ; white doubled arrow). (**c**) Laminations (black lines) and enamel prisms (black arrows) identified in the enamel of *E*. *hemionus* (IPS92347). (**d**) Laminations (black lines) between consecutive Retzius lines (black dashed lines) in the outer enamel of *E*. *grevyi* (IPS84963). (**e**) Angle between the enamel dentine junction (EDJ; white line) and the enamel formation front (EFF; white dashed line) in *E*. *quagga* (IPS92346). C = cementum; D = dentine; E = enamel. Black scale bars: 5 mm; white scale bars = 50 μm.

**Figure 2:**
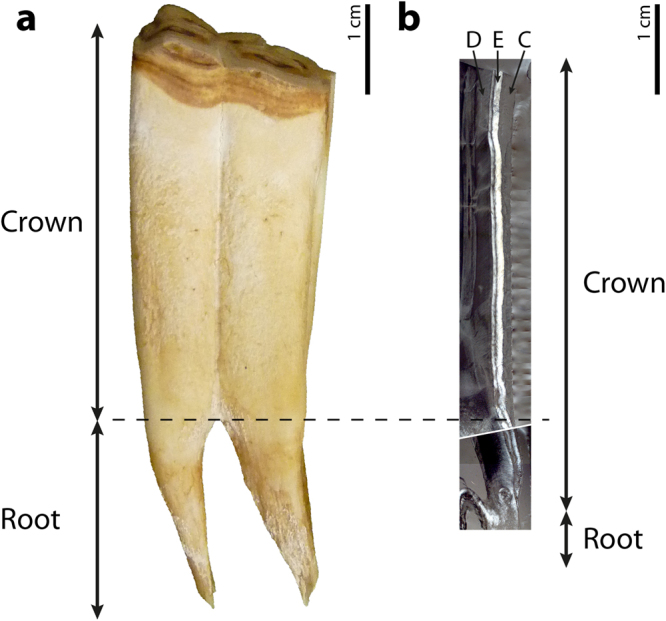
Macroscopic (**a**) *versus* histological (**b**) distinction of tooth crown and root. Figure shows that dental enamel extends beyond the limit of the macroscopic crown (black dashed line). (**a**) First lower molar of *E*. *quagga* (IPS92346) in buccal view. (**b**) Histological section of the buccal cusp in IPS92346. C = cementum; D = dentine; E = enamel.

Enamel, dentine and cementum are the three dental tissues that conform the molar crown in all extant *Equus* (Figs 1a,b; 2b). A thin layer of enamel is also observable in the region that is macroscopically considered as the root^13,32,47^ (Fig. 2). Therefore, the molar crown, understood as the part of the tooth composed of enamel^22^, extends further than macroscopically considered^47,48^ (Fig. 2). To avoid confusion between the morphological and histological distinction of tooth root in *Equus* first molars, the term root will be used here to designate the most apical area of the tooth which is enamel-free^49^ (Fig. 2b).

Laminations are fine incremental markings running parallel to the enamel formation front^20^ and are the most common incremental features identified in the enamel of our equid sample (Fig. 1c). Considering that laminations follow the one-day periodicity previously described in other herbivorous mammals^3,8,26–28^, we calculated the crown formation time (CFT) of unworn teeth. As it is shown in Table 1, CFT estimations are a reasonable match for the age of the specimen previously determined by classical methods, confirming the daily periodicity of laminations in equids.

**Table 1:** Age and crown formation time (CFT) estimates in unworn teeth of extant species.

Species	Code	CFT (days)	CFT (months)	Estimated age (months)
*E*. *hemionus*	IPS83153	137	5	0 − 6
	IPS83151	184	6	6 − 12
*E*. *quagga*	IPS92345	134	4	0 − 3
	IPS92342	160	5	3 − 6
*E*. *grevyi*	IPS84964	117	4	0 − 6

Long-termed Retzius lines, prominent lines formed at an oblique angle to the enamel prisms^23^, are also observed in the enamel of extant equids (Fig. 1d), mainly in cervical enamel. The periodicity of these features in our equid sample range from 5 to 7 days in *E*. *hemionus* and 5 to 6 days in both zebra species (*E*. *grevyi* and *E*. *quagga*) (RI, Table 2).

**Table 2:** Body mass, hypsodonty index and values of the different enamel growth parameters inferred in extant and extinct equids. BM = body mass; HI = hypsodonty index; DSR = daily secretion rate; RI = repeat interval; EER = enamel extension rate; EFFa = enamel formation front angle; N = number of observations; SD = standard deviation; Min = minimum; Max = maximum. The star (*) indicates fossil specimens.

Species	BM (kg)^42,58^	HI^58^	DSR (μm/d)		RI (days)	
			N	Mean ± SD	N	Min. − Max.
*E*. *hemionus*	230	4.76	45	16.92 ± 2.3	18	5 − 7
*E*. *quagga*	257	4.44	45	16.98 ± 1.64	12	5 − 6
*E*. *grevyi*	384	5.19	37	17.74 ± 1.6	13	5 − 6
*E*. ferus*	350	3.8	28	17.8 ± 2.48	21	5 − 7
*E*. hydruntinus*	215	3.54	28	18.87 ± 2.01	22	4 − 6
**Species**	**EER (μm/d)**					
	**CDS I**		**CDS II**		**CDS III**	
	**N**	**Mean** ± **SD**	**N**	**Mean** ± **SD**	**N**	**Mean** ± **SD**
*E*. *hemionus*	28	349.28 ± 118.7	23	123.46 ± 76.32	48	31.38 ± 11.58
*E*. *quagga*	14	427.08 ± 129.58	12	140 ± 65.56	17	28.21 ± 9.25
*E*. *grevyi*	7	358.39 ± 101.33	13	153.37 ± 81.26	17	36.83 ± 12.58
*E*. ferus*	—	—	26	138.04 ± 52.74	—	—
*E*. hydruntinus*	12	256.82 ± 122.64	21	97.40 ± 45.01	—	—
**Species**	**EFFa (degrees)**					
	**CDS I**		**CDS II**		**CDS III**	
	**N**	**Mean** ± **SD**	**N**	**Mean** ± **SD**	**N**	**Mean** ± **SD**
*E*. *hemionus*	25	1.25 ± 0.54	17	3.35 ± 0.87	17	10.63 ± 3.4
*E*. *quagga*	14	1.49 ± 0.63	9	3.34 ± 0.76	16	11.13 ± 3.85
*E*. *grevyi*	8	1.21 ± 0.3	7	2.9 ± 0.87	15	11.73 ± 3.37
*E*. ferus*	—	—	39	3.47 ± 1.46	—	—
*E*. hydruntinus*	17	2.33 ± 1.16	20	4.34 ± 2.27	—	—

**Figure 3:**
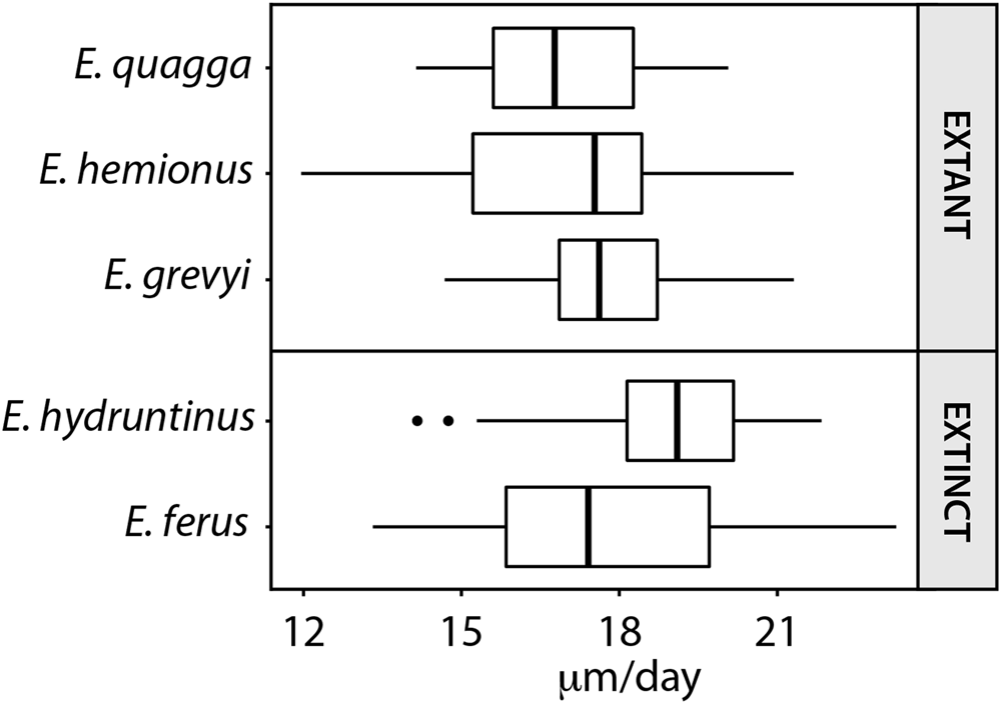
Boxplot of the daily secretion rate (DSR) of the enamel in the extant and extinct species of *Equus* analysed.

A mean daily secretion rate of ≈17–18 μm/d was calculated in the enamel of extant equids (DSR, Table 2) regardless of the part of the crown (occlusal, cervical) or the enamel zone (inner, outer) analysed (Kruskal-Wallis, p-value > 0.05, Supplementary Table S1, S2). The Asiatic wild ass shows the slowest daily secretion rate within the extant species, while Grevy’s zebra presents the fastest rate (Table 2, Fig. 3). However, no significant differences have been observed among the species analysed (Kruskal-Wallis, p-value > 0.05, Supplementary Table S3) (Fig. 3).

**Figure 4:**
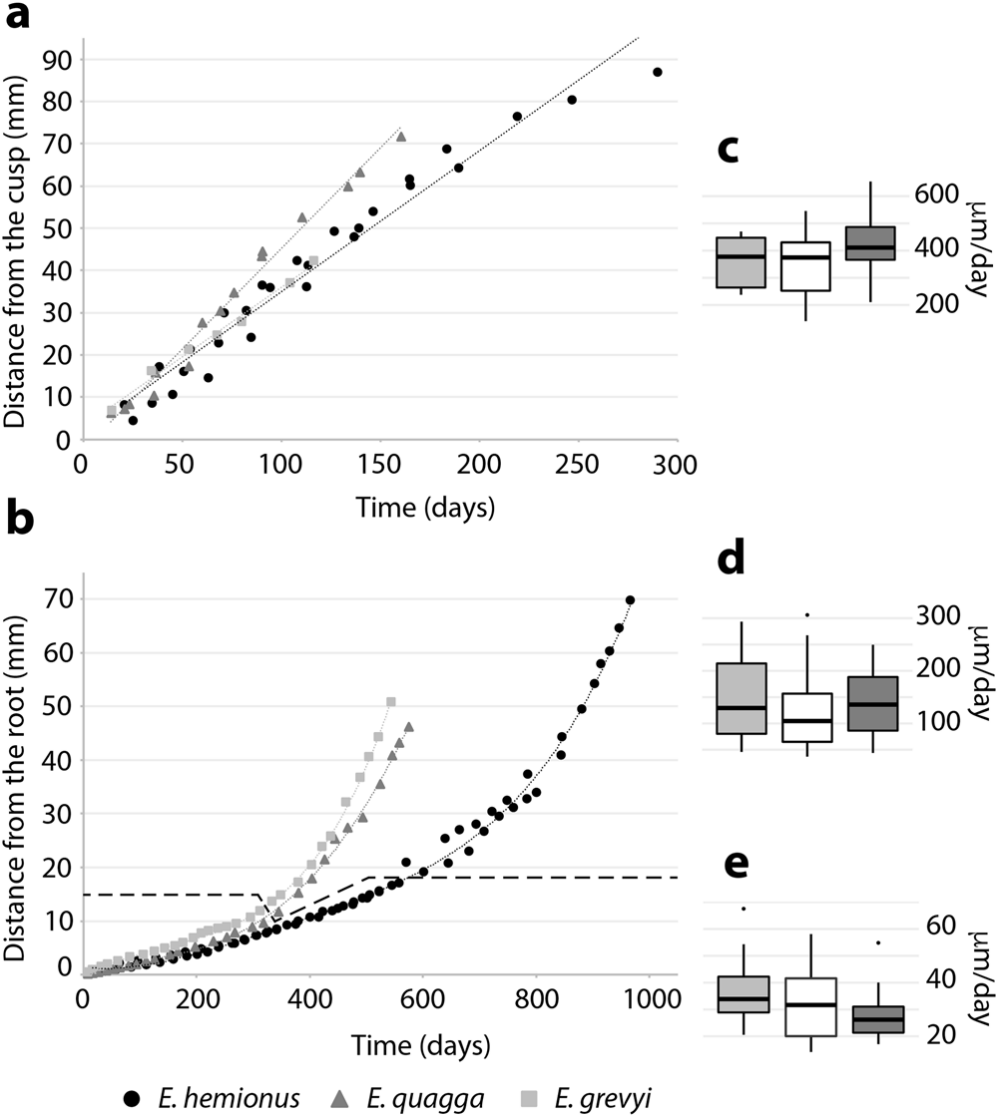
Crown formation time (CFT) related to crown height (**a**,**b**) and enamel extension rates (EER) (**c**,**d**,**e**) in the extant *Equus* species studied. (**a**) CFT related to crown height in unworn teeth. (**b**) CFT related to crown height in worn teeth. Dashed line separates measures obtained for the enamel that is deposited before (upward) and after (downward) the crown divergence. (**c**) EER of unworn teeth studied. (**d**) EER in macroscopic crowns of worn teeth. (**e**) EER of the enamel deposited beyond the limit of the macroscopic crown. Legend for scatter plots is shown at the bottom of the figure. 75% grey boxplot = *E*. *quagga*; 25% grey boxplot = *E*. *grevyi*; white boxplot = *E*. *hemionus*.

The pace of growth of the first lower molars of extant equids (crown height against crown formation time) is plotted in Fig. 4a and b. As equid teeth start to wear before the crown is completely formed^32,49^, crown development is reconstructed from the cusp tip in unworn teeth (Fig. 4a) and from the root in worn molars (Fig. 4b). Differences in growth between species have only been identified in the most cervical enamel (Fig. 4b), concretely, at the beginning of the morphological root^13,32,47^ (Fig. 2a). As Fig. 4b shows, *E*. *hemionus* deposits enamel in that part of the tooth for a longer period than both zebras. In all studied species, unworn teeth without roots experienced fast linear growth (Fig. 4a) while growth curves of worn molars fit to a polynomial of quadratic order (Fig. 4b). These results indicate that teeth experience different types and rates of growth during formation. Furthermore, the inflection point of the polynomial growth curve (Fig. 4b) is related to macroscopic anatomical changes in the tooth, as it matches the crown divergence that results in the formation of the morphological roots (Fig. 2a). Enamel, then, is still being deposited after the morphological roots are formed, but at much lower rates (Fig. 4b,e).

Three different crown developmental stages (CDS) that significantly differ in enamel extension rate (Kruskal-Wallis, p-value < 0.05, Supplementary Table S4) could be established based on the different growth patterns (Fig. 4). During a first stage of fast linear growth (Fig. 4a) enamel extends at a mean rate of 350–400 μm/d (Fig. 4c, Table 2). At the second stage of development, which corresponds to the fastest period of polynomial growth (Fig. 4b), enamel extension progressively decreases up to rates of ≈130 μm/d (Fig. 4d, Table 2). At the third stage, enamel extension rate (EER) decreases quickly to ≈30 μm/d (Fig. 4e, Table 2), representing the slowest period of polynomial growth (Fig. 4b). The decrease in the rate of enamel extension observed throughout tooth formation is mainly due to changes in the enamel formation front angle (EFFa), which presents a range of values that vary from ≈1° on the tooth cusp to ≈11° near the root (Table 2). The EFFa reflects the number of ameloblasts that are secreting matrix at the same time, with smaller angles indicating higher EERs because more ameloblasts are activated along the EEF^3,50^. Thus, the number of activated ameloblasts in the first lower molars of extant equids is progressively reduced during the development of their crowns.

**Figure 5:**
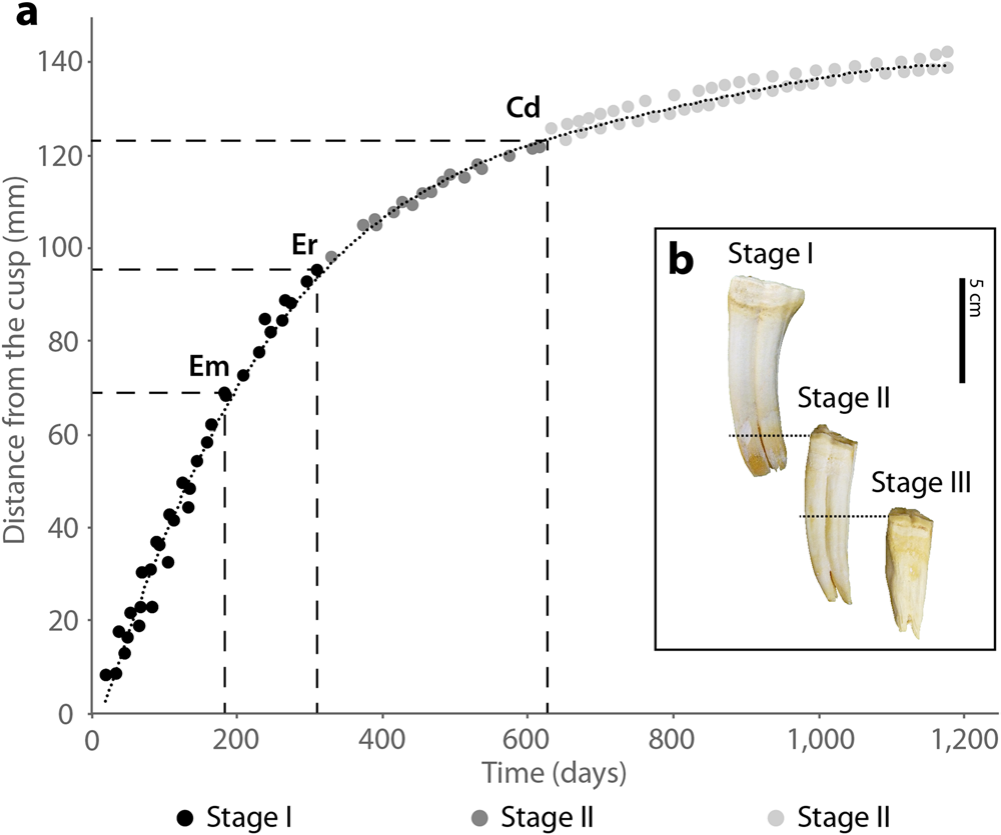
Reconstruction of growth of the first molar crown in *Equus hemionus* based on similarities in EER. (**a**) Crown formation time against crown height. Three different crown developmental stages (CDS) are identified from the slope of the curve. (**b**) Teeth of *E*. *hemionus* that differ on degree of wear and root development showing the correspondences of EER (black dashed lines) that enabled growth reconstruction (Stage I = IPS83155; Stage II = IPS92347; Stage III = IPS92339). Em = emergence; Er = eruption; Cd = crown divergence. Colour legend is shown at the bottom of the figure.

No significant differences have been found in the rate of enamel extension between the different species of extant *Equus* analysed within each CDS (Kruskal-Wallis, p-value > 0.05, Supplementary Table S5). This suggests that EER is conservative and characteristic for each developmental stage in extant *Equus*. On that basis, we reconstructed the complete growth of the crown for the first lower molars of the Asiatic wild ass (Fig. 5a), establishing the overlap area between unworn and worn teeth where similarities in EER were found (Fig. 5b). The timing of several ontogenetic and structural changes of the first molar, such as the time of emergence, eruption and crown divergence obtained from dental histology (Fig. 5a), agrees well with data in the literature on timing of occurrence of these events in equid’s first molars^32,51,52^. This confirms the validity of the growth reconstruction based on EER for this species and indicates that the complete crown of the first lower molar of *E*. *hemionus* takes about three years to be formed. Changes in curve’s slope coinciding with teeth eruption and crown divergence indicate the start of the different CDS (Fig. 5a).

We performed superimposition of teeth considering similarities in EER to infer data about wear rate in the species. As shown in Fig. 5b, the cervical portion of a recently erupted tooth from a one-year old individual (IPS83155) presents similar extension rates as the occlusal part of the crown of a seven-year-old tooth (IPS92347), while EER values at the mid-crown of IPS92347 match those estimated on the occlusal enamel of a thirteen-year-old individual (IPS92339). Superimposition of teeth indicates that approximately 110 mm of tooth crown has been worn down in 12 years: almost 80 mm during the first six years of life and around 30 mm during the next six (Fig. 5). Thus, a wear rate of around 13 mm/y is estimated for the first six-year period, while this is 5 mm/y for the second one. These results indicate that most of the crown formed during the first year of life (IPS83155, Fig. 5b) is worn away by when the individual is seven years old (IPS92347, Fig. 5b).

### Dental histology of fossil *Equus*

As in extant *Equus*, both laminations and Retzius lines are identified in the enamel of the Pleistocene fossils studied. Long-termed Retzius lines present a periodicity of 5 to 7 days in *E*. *ferus*, while they are deposited every 4 to 6 days in *E*. *hydruntinus* (Table 2).

The fossil species analysed (*E*. *ferus* and *E*. *hydruntinus*) secrete enamel at similar rates (Kruskal-Wallis, p-value > 0.05, Supplementary Table S3) (Fig. 3, Table 2). When compared with extant species, however, differences in daily secretion rate have been found between *E*. *hydruntinus*, *E*. *quagga* and *E*. *hemionus* (Fig. 3) (Kruskal-Wallis, p-value < 0.05, Supplementary Table S3). The small extinct species *E*. *hydruntinus* secretes enamel a mean rate of ≈19 μm/d, while the Plains zebra and the Asiatic wild ass do at ≈17 μm/d (Fig. 3, Table 2).

**Figure 6:**
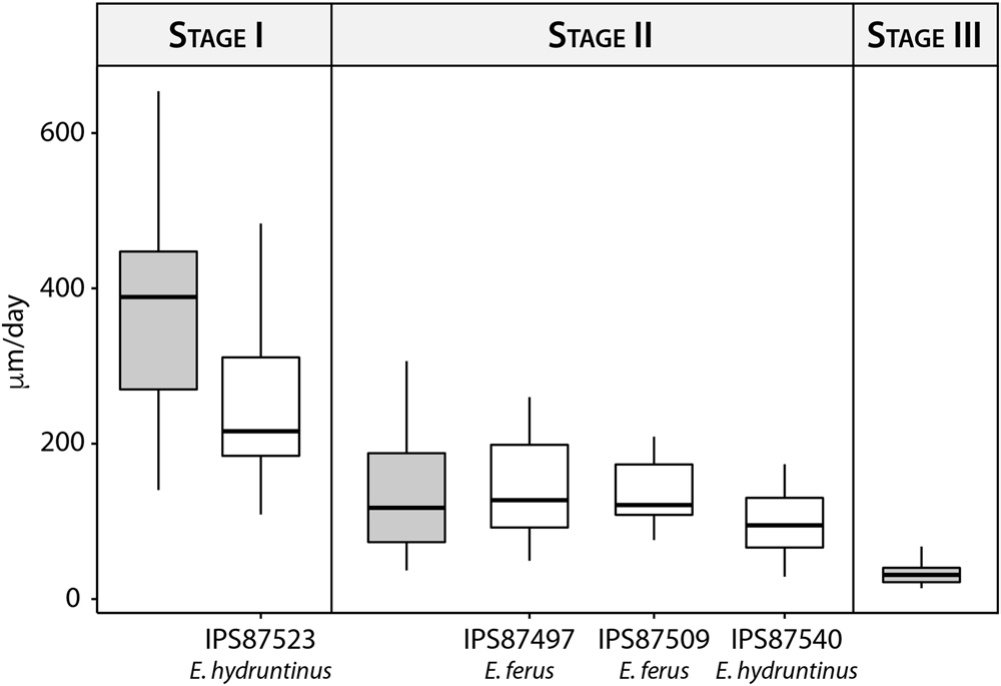
Enamel extension rate of Pleistocene *Equus* specimens compared with the mean enamel extension rate of the three crown developmental stages established for extant species (grey boxplots).

We analysed the rate of enamel extension of each fossil tooth based on its macroscopic appearance and the CDS established for extant equids (Fig. 6). The EER of IPS87523 (*E*. *hydruntinus* Stage I, Table 2) matches the first stage of crown development (Kruskal-Wallis, p-value > 0.05, Supplementary Table S6) (Fig. 6) and its macroscopic appearance (slight wear and no presence of morphological roots, Table 3) is also that expected for this developmental stage. The rate of enamel extension of IPS87540, IPS87497 and IPS87509 (*E*. *hydruntinus* and *E*. *ferus* Stage II, Table 2) corresponds to that estimated for the second phase of crown formation (Kruskal-Wallis, p-value > 0.05, Supplementary Table S3). As these teeth show moderate wear and macroscopic roots (Table 3), a stage II of development was expected for their crowns too. These results on EER of fossil *Equus* confirm that this dental growth parameter is very conservative within the genus.

**Table 3:** Sample studied. E: erupted; NE: no erupted; mo.: months; y: years; ZIHU: Zoological Institute of Hamburg University (Hamburg, Germany); ICP: Catalan Institute of Paleontology (Barcelona, Spain). The star (*) indicates fossil species.

Species	Code	Eruption stage	Wear degree	Estimated age	Site	Collection
*E*. *hemionus*	IPS83153	NE	Unworn	0 − 6 mo.	Hagenbeck Zoo	ZIHU
	IPS83151	E	Unworn	6 − 12 mo.	Hagenbeck Zoo	ZIHU
	IPS83155	E	Slight	1 − 2 y.	Hagenbeck Zoo	ZIHU
	IPS92347	E	Moderate	7 y.	Hagenbeck Zoo	ZIHU
	IPS92339	E	Advanced	13 y.	Hagenbeck Zoo	ZIHU
*E*. *quagga*	IPS92345	NE	Unworn	0 − 3 mo.	Hagenbeck Zoo	ZIHU
	IPS92342	E	Unworn	3 − 6 mo.	Hagenbeck Zoo	ZIHU
	IPS92346	E	Moderate	5 y.	Hagenbeck Zoo	ZIHU
*E*. *grevyi*	IPS84964	NE	Unworn	0 − 6 mo.	Hagenbeck Zoo	ZIHU
	IPS84963	E	Moderate	5 y.	Hagenbeck Zoo	ZIHU
*E*. ferus*	IPS87509	E	Moderate	Adult	La Carihuela	ICP
	IPS87497	E	Moderate	Adult	La Carihuela	ICP
*E*. hydruntinus*	IPS97523	E	Moderate	Adult	La Carihuela	ICP
	IPS87540	E	Slight	Subadult	La Carihuela	ICP

## Discussion

The aim of the present research was to reconstruct, for the first time, the pace of growth and development of the first lower molars in *Equus* based on the analysis of enamel microstructure. Until now, most of the research concerning enamel histology has been limited to low-crowned, brachydont teeth of different primate species^1^ and only a few studies have focused on hypsodont mammals^3,8,28,53^. Our findings in three species of extant (*E*. *hemionus*, *E*. *quagga* and *E*. *grevyi*) and two Pleistocene (*E*. *ferus* and *E*. *hydruntinus*) equids contribute to the knowledge of enamel microstructure in high-crowned vertebrates and provide the basis for future research in Perissodactyls. However, the results obtained here are not only relevant for histological research but also for isotopic studies on fossil and archaeological vertebrates. *Equus*’ teeth, concretely equid enamel, are widely used in palaeobiological and palaeoecological isotopic studies^15,17,18^ because their extremely high crowns record several years of the individual’s life^13^. A thorough understanding of the timing, geometry and rate of enamel maturation is key to interpretation of isotopic results^54^. Although enamel incremental marks do not provide relevant information about the timing^26^ and/or the pattern^54^ of enamel mineralization because they only register the secretory stage of amelogenesis^20^, these features are known to yield accurate estimations on tooth growth rates^19^. Indeed, our results on enamel extension rates (≈130 μm/d or ≈48 mm/y for the second CDS, Table 2) are similar to the maturation rates reported for equids (40–60 mm/y)^55^. Thus, rates of enamel extension seem to be a good proxy of rates of enamel maturation in *Equus*. This finding, along with the detailed description of the growth and development of the equid crown presented here, will help in the understanding of the isotopic microsamples extracted from this mammalian group^12,13^.

### Crown height and formation time

To date, the timing and pace of crown formation in equid teeth has been assessed by measuring crown height^12,35^ and by identification of dental roots in radiographic images^13,32–34^. In agreement with previous studies^47,48^, our results indicate that the external division of teeth into crown and root does not match the histological definition of these structures (Fig. 2), as dental enamel extends over the limit of what is usually considered the macroscopic crown of the tooth^47^ (Fig. 2b). As enamel is characteristic of a tooth’s crown^22^, we suggest that this portion of the tooth should be considered part of the crown and not part of root; this view is in contrast to a previous study by Kirkland *et al*.^32^. Apart from this terminological issue, it is worth noting that the presence of enamel beyond the crown divergence might affect results of previous studies which considered this anatomical point to be the crown’s end. Thus, reconstructions of crown growth that exclude its final portion^12,13^ may underestimate total crown formation time, as this cervical enamel takes up to one or two years to be formed in zebras and *E*. *hemionus*, respectively (Fig. 4b), due to low rates of enamel extension (Fig. 4e, Table 2). On the other hand, the discrepancy between macro- and microanatomical tooth structure (Fig. 2) may lead to a miscalculation of dental indexes that involve measurements of the complete crown. For instance, the hypsodonty index usually used in palaeoecological studies^47,56^ requires the identification of the crown end to measure the crown height^57^. Consequently, difficulty in differentiating the exact point where enamel ends from tooth macroanatomy^47^ (Fig. 2) makes the estimation of the degree of hypsodonty in *Equus* a challenging issue. Thus, hypsodonty values previously reported for this genus^57–59^ should be viewed with caution because the crown-root transition might be not homologous when measuring crown height. Furthermore, the enamel identified in the morphological roots represents up to 1–1.5 cm of tooth height (Fig. 4b). Failure to consider this area when measuring crown height leads to underestimation of the hypsodonty index.

An alternative approach to estimating hypsodonty in equids can be made from enamel histology. Our results indicate a longer period of crown formation in *E*. *hemionus* than in both African zebra species (Fig. 4b), suggesting a higher hypsodonty in the former species. However, previous studies described similar hypsodonty values for these three equids^57–59^. The increase in crown height that characterises hypsodont teeth has usually been explained as an adaptation which extends the durability of the teeth in animals feeding on an abrasive diet^56,57^. Like all extant equids, *E*. *hemionus*, *E*. *quagga* and *E*. *grevyi* are classified as grazers^38–41,60^ with similar abrasive diets^60^. However, they dwell in different habitats, which also influence tooth wear^61^. While the Asiatic wild ass is endemic on the steppe and desert plains of central Asia^38,40^, *E*. *quagga* and *E*. *grevyi* usually occur in different types of African grasslands^38,41^. Our findings of an extended deposition of enamel in the Asiatic wild ass (Fig. 4b) is congruent with previous research that correlates hypsodonty and mean annual precipitation^60,61^, as this species dwells in a more arid habitat. Furthermore, hypsodonty has been explained to occur in response to an increase in lifespan of the species^56,62–64^. A higher degree of hypsodonty in *E*. *hemionus* (Fig. 4b) is consistent with this hypothesis, as the maximum longevity reported for this species in the wild is 29 years^65^, while it is 18 and 21 years for Grevy’s zebra^66^ and plains zebra^51^, respectively. Theory suggests that in a resource-limited environment with low extrinsic mortality, natural selection favours a shift in energy allocation from reproduction to growth and maintenance^67^, and that triggers extended longevity^62,64,68,69^. The Asiatic wild Ass inhabits a resource-poor environment (steppes and deserts of Asia^40^) and faces low rates of predation (grey wolves are its only known non-human predators^70^). Thus, the increase in crown height observed in *E*. *hemionus* can alternatively be explained as an increase in durability of its teeth to extend life span. The extended deposition of enamel in the Asiatic wild Ass has been identified after the crown divergence, at the beginning of the macroscopic root (Fig. 4b). Although, as far as we know, there are no descriptions of equid molars which are worn below the crown bifurcation, teeth with such exceptional degree of wear could still be functional. In very old horses, teeth are known to present minimal reserve crowns and very elongated roots that allow stable alveolar attachment^49^. Thus, a hypothetical equid molar worn below the crown divergence would be viable. This also supports the hypothesis of a prolonged enamel deposition in *E*. *hemionus* related to the extended longevity of the species, as the presence of enamel beyond the crown bifurcation increases the durability of the tooth in animals of advanced ontogenetic stages.

### Crown developmental stages (CDS)

Our results indicate that the rate of crown formation exponentially decreases throughout tooth development (Figs 4a,b; 5), as already suggested by Bendrey *et al*.^12^. The EER decreases from values of ≈350–400 μm/d at the beginning of crown development to rates of ≈30 μm/d at the end of crown formation (Fig. 4c–e, Table 2). A reduction on EER during crown development has also been observed in other mammalian species^1,8,10,28,31^. Based on such variation of EER along the tooth’s crown, we determined three different developmental stages of the crown (CDS) in the first lower molars of extant *Equus* (Fig. 4, Table 2). During a first phase of crown formation, dental enamel grows fast and linearly (Fig. 4a,c, Table 2). The next stages of enamel development, however, follow polynomial growth (Fig. 4b). Thus, the second CDS, which presents extension rates values of around ≈130 μm/d (Fig. 4d, Table 2), corresponds to the fastest period of this polynomial growth (Fig. 4b), while CDS III represents the slowest period (Fig. 4b) with very low rates of enamel extension (Fig. 4e, Table 2). The limit between the different CDS seems to correlate with both structural and ontogenetic changes in the tooth in *E. hemionus* (Fig. 5a). Therefore, the transition from stage I to stage II matches the first molar’s eruption time^52^, while the beginning of stage III correlates well with the macroscopic appearance of the tooth roots in related *Equus* species^32^ (Fig. 5a). Correspondence between the beginning of CDS III and the divergence of the crown has also been detected in *E*. *quagga* and *E*. *grevyi* (Fig. 4b). Our results also show that the rate of enamel extension is very conservative within the genus *Equus* and, as previously mentioned, characteristic for each CDS (Figs 4c–e; 6). This result is especially relevant for palaeodemographic studies, as EER estimations of a fragmentary fossil/archaeological equid tooth allow researchers to assigned it to a CDS and, hence, to an age category. Studies aimed to infer the life history strategy of extinct species, however, require combined analysis of the macroscopic appearance of the tooth and the microscopic examination of its EER. Thus, in the case of the estimated CDS of a fossil tooth failing to match the degree of dental wear and/or root development expected for this stage, differences in growth and, thus, in life history might be deduced for the species.

### Estimation of wear rates

Superimposition of teeth of *E*. *hemionus* based on similarities of EER (Fig. 5) also yield data on wear rates in *Equus*. A wear rate of around 13 mm/y has been estimated for the first six years of life, a value far above the 3–5 mm/y previously reported in extant^49,71^ and in extinct equids^72^. However, the latter published wear rates agree with the estimated wear rate of 5 mm/y obtained for the next six-year period. Therefore, our results suggest that (i) first lower molars of the Asiatic wild ass wear down at an exponentially decreasing rate and that (ii) wear rates are much higher at the beginning of the animal’s life, as already proposed by Levine^35^. This is explained by the eruption sequence of the species. As the first molar is the first permanent tooth to erupt, and complete permanent dentition is not visible until almost the fifth year of life^52^, it seems reasonable that higher wear rates in the first molar would be found at early ontogenetic stages. Due to these greater wear rates, the crown formed during the first year of life is almost worn down in a seven-year-old tooth (Fig. 5b). This observation is of special interest for the correct interpretation of isotopic values obtained from enamel microsampling in equids.

### Daily Secretion Rate (DSR)

The DSR of enamel in equids has recently been a matter of discussion^28,31^. The results obtained here suggest that extant equid species secrete enamel at a mean rate of ≈17–18 μm/d (Fig. 3, Table 2). These values agree well with rates of enamel apposition reported for other hypsodont mammals^3,8,27,28^, but they differ from previously published estimates of only 5 μm/d for the domestic horse^13^. Kierdorf *et al*.^29,31^ considered that these lower values of enamel apposition rates reported in horses might be due to a misidentification of sub-daily incremental marks as daily marks. The daily periodicity of laminations is well-established in several mammalian taxa using experimental labelling^23,28,31^, but this type of study has never been conducted in equids. Therefore, we compared the estimated age of the youngest individuals with the time of formation of their first molar crown (Table 1), in order to validate and corroborate the periodicity of these features in *Equus*. As the formation of the first permanent molar in equids starts around the time of birth^13,33^, the CFT of the still-developing, unworn tooth must be equivalent to the previously calculated age of the individual. Our results show that both methodologies provide similar times for crown formation (Table 1). These results confirm the hypothesis of Kierdorf *et al*.^29,31^ that previous studies misidentified incremental lines in equid enamel^13^.

### First insights into enamel histology of fossil equids

Finally, the enamel microstructure of the Late Pleistocene species, *E*. *ferus* and *E*. *hydruntinus*, was analysed in a first attempt to infer their pattern of molar growth. Both fossil equids present similar values for enamel secretion and extension rate but differ in the periodicity of Retzius lines, namely the repeat interval (RI) (Table 2). As is shown in Table 2, RI in *E*. *hydruntinus* comprises 4–6 days, while RI in *E*. *ferus* consists of 5–7 days. In agreement with previous research on primates and proboscideans^73^, these results suggest that equid repeat interval is related to body mass, as *E*. *hydruntinus* presents the lowest body mass as well as the lowest RI within the equid species investigated. The EER of all fossil teeth studied was as expected due to their macroscopic appearance (degree of wear and root development) (Fig. 6), which suggests that the time of crown formation and eruption in both extinct species was similar to that reported for extant equids. When comparing extant and extinct *Equus*, our results show that the daily secretion rate of enamel in *E*. *hydruntinus* significantly differs from that of *E*. *quagga* and *E*. *hemionus* (Fig. 3, Table 2). According to Dirks *et al*.^10^, “the daily secretion rate of enamel is likely to be dependent on a complex interaction of tooth size, morphology and life history”. A full assessment of the factors responsible for the higher rates of enamel apposition detected in *E*. *hydruntinus* requires a more detailed analysis involving a larger sample size, beyond the scope of the present study. However, some hypotheses can be drawn from our results. On the one hand, our findings do not match expected DSR values for this species based on body size (life history factor) or hypsodonty (tooth size factor). As *E*. *hydruntinus* is the smallest and less hypsodont equid analysed here (Table 2), an enamel secretion rate comparable to that of *E*. *quagga* would be expected. The unexpected elevation in daily secretion rates in this extinct equid species might be related to tooth morphology. Although genetically related to the extant Asiatic wild ass, *E*. *hydruntinus* is a singular extinct species that shares morphological features with extant asses, zebras and the Pliocene equid, *E*. *stenonis* ^74–76^. Specifically, the lower molar enamel of *E*. *hydruntinus* presents a primitive pattern that appears to be similar to that of *E*. *stenonis* ^75^. Future histological studies of the enamel in this early Pliocene species might shed light on the factors that lead to the daily secretion rates found in *E*. *hydruntinus*.

In conclusion, our histological analysis of enamel in the first lower molars of several extant and extinct *Equus* species allowed the estimation of dental growth parameters and the reconstruction of the enamel growth pattern in the clade. Our results provide further evidence of the already known discrepancy between dental macro- and microanatomy that might hamper growth inferences using external measurements of the crown. This finding calls for a revision of commonly used palaeontological and palaeoecological indexes, requiring a correct identification of the crown-root transition, such as the hypsodonty index. We identified three crown developmental stages (CDS) based on different growth patterns during the formation of the first molar. The beginning of each CDS is related to ontogenetic and morphological changes of the tooth. EER is very conservative within the genus and characteristic for each of the three CDS, thus showing a high potential for use in palaeodemographic and/or life history studies. We estimated a daily secretion rate for enamel of ≈17–18 μm/d in the three extant *Equus* species, suggesting that previously reported values of DSR in *E*. *caballus* are erroneous. Superimposition of different-aged teeth of *E*. *hemionus* reveals a high wear rate during the first six years of the animal’s life. This information should be considered in isotopic studies of tooth enamel to correctly interpret isotopic microsamples. Our results show that there are no differences in extension rate or daily secretion rate in the enamel of *E*. *hemionus*, *E*. *quagga* and *E*. *grevyi*. However, the Asiatic wild ass shows a longer period of crown formation in comparison with plains zebra and Grevy’s zebra, which indicates a higher hypsodonty in the ass. Such increase in tooth height is likely to be an adaptation of the Asiatic wild ass to counterbalance tooth wear resulting from both the more arid habitat and the extended longevity of the species. Finally, enamel histology of the Pleistocene species (*E*. *ferus* and *E*. *hydruntinus*) reveals that the smallest fossil species secreted enamel at higher rates than extant equids of the same size, *E*. *quagga* for example. We suggest that this might be related to the tooth morphology of *E*. *hydruntinus* rather than its tooth size or life history. Further studies with a larger sample size and related taxa are needed to corroborate these results.

## Methods

### Material

In the present study, we analysed 14 first lower molars from three extant and two Pleistocene species of *Equus* (Table 3). A total of 10 teeth representing different eruption stages and degrees of wear were studied in specimens from three extant species (*E*. *hemionus*, *E*. *grevyi* and *E*. *quagga*) from the Hagenbeck Zoo (Hamburg, Germany). Each extant specimen was aged by the eruption and wear patterns described for the different *Equus* species^51,52,77,78^. In view of this methodology becoming less reliable when all permanent teeth are erupted, the age of adult individuals was confirmed by counting annual growth marks present in the cementum of the first lower incisors^51,52^. In addition to the extant samples, four teeth of the Pleistocene species, *E*. *ferus* and *E*. *hydruntinus*, were examined. Each fossil tooth was assigned to an age category (sub-adult or adult) based on the degree of wear and root development^32^. The fossil material came from La Carihuela, a Late Pleistocene cave located in Piñar (Granada, Spain) and dated between 82,500 and 11,200 years BP^79–81^. Fossil samples were housed at the Catalan Institute of Palaeontology (Barcelona, Spain), while extant specimens belonged to the collections of the Zoological Institute of Hamburg University (Hamburg, Germany).

### Preparation of histological thin-sections

Histological sections of teeth were prepared in our laboratory following standard procedures^8^. In the case of extant species, teeth were firstly extracted from the mandible and dehydrated using different concentrations of alcohol for a total period of 72 hours (70, 96 and 100%; 24 h in each). With both extant and fossil samples, each tooth was then embedded in epoxy resin (Araldite 2020) and longitudinally sectioned at the level of the protoconid in the bucco-lingual plane using a low-speed diamond saw (IsoMet, Buehler). The cut surface was later polished using a Metaserv®250 (Buehler) and fixed to a frosted glass with ultraviolet-curing adhesive (Loctite 358). Each sample was then cut and ground with a diamond saw (PetroThin, Buehler) up to a thickness of 150 μm and polished again to obtain a final thickness of approximately 120 μm. Finally, the thin sections obtained were dehydrated again in increasing concentrations of alcohol, immersed in a histological clearing agent (Histo-Clear II) and mounted using DPX medium (Scharlau) to improve visualisation of the dental microscopic features. Due to the high height of equids’ crowns, most of the samples had to be mounted on two separate slides^10^. The identification of incremental markings enabled confirmation of both slides being cut from the same plane (Fig. 1a).

### Analysis of thin histological sections

Thin sections were examined using a polarised light microscope (Zeiss Scope.A1 microscope) and images were captured with a digital camera mounted on the microscope (AxioCam ICc5). Enamel tissue was examined in detail and different types of incremental markings were identified. Counts and measures of daily periodic laminations (Fig. 1c) along the buccal cusp were performed using ImageJ software, allowing estimates of several histological parameters that reflect the growth rate of the enamel. Firstly, the daily secretion rate (DSR) was calculated in different areas of the crown by measuring the distance between adjacent daily lines following the course of enamel prisms^8^ (Fig. 1c). Secondly, the repeat interval (RI), representing the periodicity of long-period lines, was quantified by counting the number of laminations between Retzius lines^1^ (Fig. 1d). In those areas where laminations were not well-preserved, this parameter was estimated by dividing the distance between consecutive Retzius lines by the DSR^1^. Thirdly, the course of incremental marks was used to reconstruct the crown formation time (CFT) of each tooth, following the method of Jordana & Köhler^8^. As indicated in Fig. 1b, the path of the incremental lines was traced from the enamel-dentine junction (EDJ) to the enamel surface. The distance between these lines was then measured following the course of the enamel prism; this value was divided by the DSR to determine the time required to form a specific portion of EDJ. The sum of all times along the EDJ results in the CFT. In unworn teeth of extant species, in which the crown development has not been completed, the CFT was compared with the estimated age of each specimen^51,52^ to validate and calibrate the daily periodicity of laminations in *Equus*. Fourthly, the enamel extension rate (EER) was calculated as the quotient between a determinate length of the EDJ and the number of days that it takes to be formed, as this parameter represents the growth of teeth along the EDJ^1^. The angle between the enamel formation front (EEF) and the EDJ was also quantified (Fig. 1e) because it is directly related to the EER^3,21^.

### Statistics

Statistical analyses were carried out with Java Gui for R© version 1.7-16^82^. Kruskal-Wallis and Mann-Whitney U tests were performed to analyse differences between groups. A value of *p* < 0.05 was considered to be statistically significant after applying the Bonferroni correction.

### Data availability

All data generated or analysed during this study are included in this published article (and its Supplementary Information files).

## Electronic supplementary material

Supplementary Material
