# Supplementary material for: Reconstructing molar growth from enamel histology in extant and extinct Equus
Source: Sci Rep. 2017 Nov 21;7:15965. doi: 10.1038/s41598-017-16227-2 (PMC5698294; doi:10.1038/s41598-017-16227-2)
Supplement: Supplementary file 1 — Supplementary Material [file 41598_2017_16227_MOESM1_ESM.pdf]

Supplementary Tables for

Reconstructing molar growth from enamel histology in extant and extinct *Equus*

Carmen Nacarino-Meneses, Xavier Jordana, Guillem Orlandi-Oliveras & Meike Köhler

Supplementary Table S1. Daily secretion rate in different parts of the crown (occlusal, middle, cervical) and enamel zones (inner, middle, outer) in the extant *Equus* studied.

Part of the crown

	Occlusal		Middle		Cervical	
	**N**	**Mean±SD**	**N**	**Mean±SD**	**N**	**Mean±SD**
E. hemionus	30	17.53±1.68	6	17.42±3.04	6	15.62±1.96
E. quagga	26	17.31 ±1.56	12	16.04±1.13	7	17.39±2.18
E. grevyi	19	18.12±1.82	12	17.52±1.24	6	17.02±1.33

Enamel zone

	Inner		Middle		Outer	
	**N**	**Mean±SD**	**N**	**Mean±SD**	**N**	**Mean±SD**
E. hemionus	17	16.18±2.6	15	17.58±2.03	13	17.11±2.06
E. quagga	14	16.98±1.82	15	16.42±1.39	16	17.5±1.6
E. grevyi	15	17.43±1.3	14	17.89±1.86	8	18.08±1.70

Supplementary Table S2. Results of Kruskal-Wallis test on differences of daily secretion rate (DSR) between enamel zones (inner, middle, outer) and between parts of the crown (occlusal, middle, cervical). P-value adjustment method: Bonferroni. $\alpha = 0.05$.

	**Chi-squared**	**p-value**
Enamel zones	2.0576	0.3574
Part of the crown	3.8434	0.1463

Supplementary Table S3. Results of Kruskal-Wallis test on differences of daily secretion rate (DSR) between *Equus* species. Pairwise comparisons were calculated using Mann-Whitney U test. P-value adjustment method: Bonferroni. $\alpha = 0.05$.

	Chi-squared	p-value
DSR	18.83402	< 0.001

	E. ferus	*E. grevyi*	*E. hemionus*	*E. hydruntinus*
E. grevyi	1	-	-	-
E. hemionus	1	1	-	-
E. hydruntinus	0.86813	0.05080	0.00265	-
E. quagga	1	0.52253	1	< 0.001

Supplementary Table S4. Results of Kruskal-Wallis test on differences of enamel extension rate (EER) between crown developmental stages (CDS) in the different extant *Equus* species analysed. Pairwise comparisons were calculated using Mann-Whitney U test. P-value adjustment method: Bonferroni. $\alpha = 0.05$.

	Chi-squared	p-value
E. hemionus	78.0673	< 0.001
E. quagga	36.5513	< 0.001
E. grevyi	28.8870	< 0.001

E. hemionus

	CDSI	CDSII
CDSII	< 0.001	-
CDSIII	< 0.001	< 0.001

E. quagga

	CDSI	CDSII
CDSII	< 0.001	-
CDSIII	< 0.001	< 0.001

E. grevyi

	CDSI	CDSII
CDSII	0.0015	-
CDSIII	< 0.001	< 0.001

Supplementary Table S5. Results of Kruskal-Wallis test on differences of enamel extension rate (EER) between species within each crown developmental stage (CDS). α = 0.05.

	**Chi-squared**	**p-value**
CDS I	2.5601	0.2780
CDS II	2.0468	0.3593
CDS III	4.3409	0.1141

Supplementary Table S6. Results of Kruskal-Wallis test on differences of enamel extension rate (EER) between Pleistocene *Equus* specimens and crown developmental stages (CDS) established for extant equids. Pairwise comparisons were calculated using Mann-Whitney U test. P-value adjustment method: Bonferroni. α = 0.05.

	**Chi-squared**	**p-value**
EER	191.3405	< 0.001

	CDS I	CDS II	CDS III	IPS87497	IPS87509	IPS87523
CDS II	< 0.001	-	-	-	-	-
CDS III	< 0.001	< 0.001	-	-	-	-
IPS87497	< 0.001	1	< 0.001	-	-	-
IPS87509	< 0.001	1	< 0.001	1	-	-
IPS87523	0.15828	0.01037	< 0.001	0.30434	0.04284	-
IPS87540	< 0.001	1	< 0.001	1	0.71513	< 0.001
